# Mass Spectrometric Study of Photoionization. I. Apparatus and Initial Observations on Acetylene, Acetylene-*d*_2_, Benzene, and Benzene-*d*_6_*

**DOI:** 10.6028/jres.068A.040

**Published:** 1964-08-01

**Authors:** Vernon H. Dibeler, Robert M. Reese

## Abstract

A windowless vacuum ultraviolet monochromator and mass spectrometer are combined for the study of photoionization processes in the energy range 2000 to 600 A (6 to 21 eV). Details of the apparatus and techniques of operation are given and results are reported for an initial study of acetylene, acetylene-*d*_2_, benzene, and benzene-*d*_6_. Ionization energies of 11.406 and 11.416 eV are obtained for the 1*π_u_* electron of C_2_H_2_ and C_2_D_2_, respectively. Vibrational levels of the ground state of the ion are observed with quantum intervals of 1855 cm^−1^(C_2_H_2_) and 1775 cm^−1^(C_2_D_2_). Ionization energies for the *e*_1_*_g_*(*π*) electron of C_6_H_6_ and C_6_D_6_ are determined to be 9.24_2_ and 9.24_5_ eV, respectively. Quantum intervals for vibrational levels of the ground state ions are apparently equal for the two isotopic molecules and estimated to be 800 cm^−1^. A second onset of ionization is observed at 11.5_3_ eV for C_6_H_6_ and at 11.5_9_ eV for C_6_D_6_. Results agree well with spectroscopic data.

## 1. Introduction

Nearly monochromatic photon beams have been used for many years [1]^1^ to study photoionization processes in low-pressure gaseous systems. The recent addition of mass spectrometric techniques [2,3] to identify unequivocally the ions produced in the photoionization process has obviated any uncertainty in the chemical composition and has shown that, at least in principle, considerable information can be obtained on the upper energy states of polyatomic molecules and the dissociation processes of ions [4].

In surmounting the experimental difficulties of combining vacuum ultraviolet spectroscopy and mass spectrometry, Hurzeler et al. [2] used a lithium fluoride window to isolate a hydrogen-discharge photon source from the high-vacuum regions of the monochromator and mass spectrometer. Thus, although the photon source included a broad energy range, the absorption of the window limited the measurements to wavelengths longer than 1040 Å (*E*<11.9 eV).

Weissler et al. [3] used no windows in their apparatus but employed a repetitive low-pressure spark to produce a widely spaced line spectrum. For some of the molecules studied, the lack of a sufficient number of emission lines in the vicinity of an onset of ionization prevented establishing that onset to better than ±30 Å (±0.5 eV). Also, the presence in the mass spectrometer of background gases from the photon source resulted in undesirable effects on ion formation.

A combined mass spectrometer-vacuum ultraviolet monochromator designed to avoid the above difficulties was recently completed and is being applied to the study of photoionization processes in the energy range 2000 to 600 Å (6 to 21 eV). Details of the arrangement, the method of operation and the results of some observations on acetylene and benzene are given in the following sections.^2^

## 2. Experimental Details

**Figure 1:**
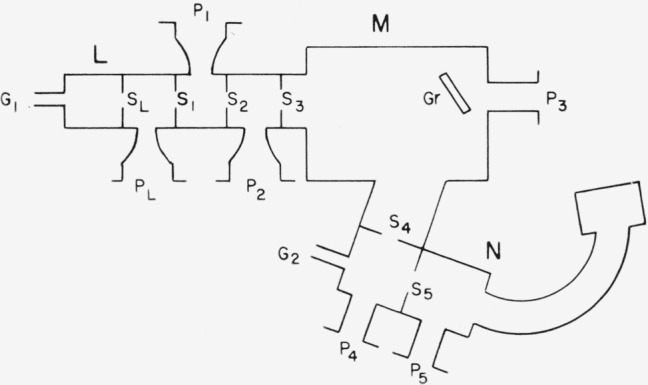
Schematic diagram showing the arrangement of photon source (*L*), monochromator (*M*), mass spectrometer (*N*), slits (*S*), and pumping ports (*P*). *S_L_*, photon source slit, 0.3×6 mm; *S*_1_, *S*_4_, entrance and exit optical slits, 0.1×10 mm; *S*_2_, *S*_3_, differential pumping slits, 2×4 mm and 4×5 mm, respectively; *S*_5_, ion defining slit, 0.5×10 mm. *P_L_*, 8 l/s mechanical pump; *P*_1_, 400 l/s Heraeus- Roots, 80 l/s forepump; *P*_2_, 700 l/s oil diffusion, 80 l/s forepump; *P*_3_, 1400 l/s oil diffusion, 40 l/s forepump; *P*_4_, *P*_5_, liq. N_2_ traps, Hg diffusion and forepumps.

Measurements were made by means of the apparatus shown schematically in figure 1. A photon source (*L*), a vacuum ultraviolet monochromator (*M*), and a mass spectrometer (*N*) are combined essentially in the manner first described by Hurzeler et al. [2]. An important exception, however, is the complete absence of absorbing windows to isolate high pressure regions from the monochromator and mass spectrometer. Thus, the entire vacuum ultraviolet region is accessible.

### 2.1. Monochromator

The Seya-Namioka type [5] instrument with a 1-m focal length was built to specifications by the McPherson Instrument Co., Acton, Mass. The photon exit arm to which the mass spectrometer is appended is nonmagnetic stainless steel. Usual materials are used elsewhere. Three optical entrance slits, sized 50, 100, and 500 *μ*, are mounted on a turret located at *S*_1_ (fig. 1) which can be rotated from outside the vacuum.

The concave, tripartite grating (*Gr*) supplied by Bausch and Lomb contains 600 grooves per mm in a ruled area 56×96 mm. It is blazed for 1500 A and coated with magnesium fluoride. With the grating in place, the first-order dispersion of the monochromator is stated by the manufacturer to be 16.6 Å/mm. The grating is rotated manually or at any one of 12 scanning speeds by a synchronous motor. The wavelength is read from a counter coupled directly to the grating drive. Adjustments are provided for correcting the linearity and the calibration of the scale. Wavelengths can be selected manually to better than 1 Å when traveling in one direction.

The monochromator is provided with two differential pumping ports (*P*_1_, *P*_2_) separated by slits (*S*_2_, *S*_3_), and located immediately behind the entrance optical slit (*S*_1_). *P*_1_ is evacuated by a 400 l/s (at 1 torr)^3^ Heraeus-Roots pump; *P*_2_, by a 700 l/s (at 10^−3^ torr) oil-diffusion pump. Each is backed by an 80 l/s (free air) mechanical pump. The grating chamber is evacuated (*P*_3_) by a 1400 l/s (at 10^−3^ torr) oil-diffusion pump backed by a 40 l/s mechanical pump. Freon or water-cooled baffles, suitable gate valves, and vacuum gages are provided at each port. Seals in high-vacuum regions are made with O-rings fabricated of fluorocarbon elastomer. Other O-rings are made of Buna-N.

The base pressure of the monochromator is about 3 × 10^−7^ torr. With hydrogen at a nominal pressure of 10 torr flowing into *G*_1_ and with slit sizes as indicated in figure 1, the system maintains a pressure differential of 10^5^ between photon lamp and monochromator chamber. With the lamp operated at higher pressures (>100 torr), additional pumping is provided at *P_L_* by means of an 8 l/s (free air) mechanical pump. This results in a pressure differential of 10^6^ between lamp and monochromator.

### 2.2. Mass Spectrometer

Analysis and detection of the photoions is accomplished by means of a conventional 15-cm radius of curvature, single focusing, sector-field instrument fabricated of Inconel and stainless steel. All vacuum seals are gold wire O-rings. The ion-source housing and analyser tube are separated by the ion defining slit (*S*_5_) and are separately pumped through 5-cm gate valves, by liquid nitrogen traps and mercury diffusion pumps. The untrapped speed of the diffusion pumps is reported by the manufacturer to be 50 l/s at 10^−3^ torr. Under conditions indicated above, the pressure differentials between lamp and ion source and between lamp and analyser tube are of the order of 10^8^ and 10^9^, respectively. Thus with a lamp pressure of 400 torr, the pressure in the analyser tube of the mass spectrometer is about 4 × 10^−7^ torr.

The ion-source housing and analyser tube are supported by the photon exit arm of the monochromator. Locating pins in the vacuum flanges maintain appropriate alinement of the photon beam, ion source, and analyser. The optical exit-slit mount is electrically insulated from the monochromator. The slit assembly is attached to the ionization chamber by screws thereby forming one wall of the chamber. The slit assembly (and ionization chamber) is attached to the mount by means of a self-locating dovetail connection. Interchangeable slit assemblies to match the entrance optical slits are available.

**Figure 2:**
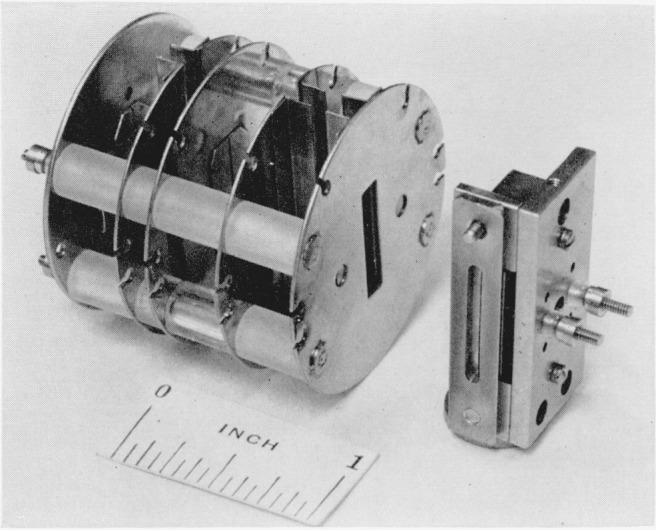
Ionization chamber and ion accelerating plates. The photon slit in the wall of the ionization chamber is just visible in the dovetailed mount. Two threaded rods are the ion-repeller connections.

Figure 2 is a photograph of the detached ion source showing the ionization chamber and the ion accelerating and focusing plates. The dovetailed exit optical-slit assembly forming one wall of the ionization chamber is visible in the right foreground. The sample inlet tube and a filament assembly and anode used for electron impact measurements are removed for the photograph.

**Figure 3:**
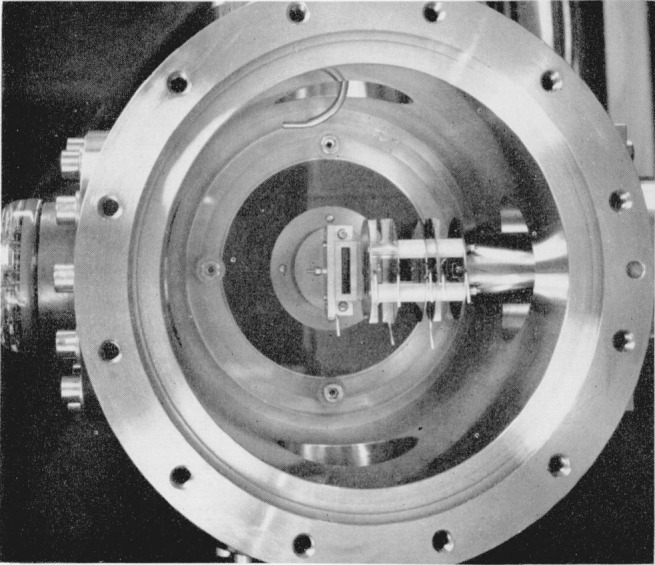
Ion-source housing with cover removed to show the mounted ionization chamber and ion accelerating plates. The photon beam leaves the ion chamber by the large rectangular slit and strikes the detector mounted on the cover plate. Photoions are accelerated toward the right of the figure. The pumping port at the bottom of the housing and the gas-inlet tube at the top are visible. In use, a flexible Teflon tube connects the gas-inlet tube with the ionization chamber.

Figure 3 shows the ion-source housing with the cover plate removed and the source mounted in place. The direction of the photon beam is toward the observer and approximately perpendicular to the plane of the photograph. The large slit in the center permits the diverging photon beam to leave the ionization chamber without striking metal surfaces until it reaches the photon monitor mounted on the coverplate. The photoions are accelerated out of the ionization chamber and toward the right of the figure. The chosen geometry of the system (orientation of photon slit relative to ion slits) results in ease of construction and alinement. Unfortunately, it also results in a smaller usable ionization volume compared with that in the geometry used by Hurzeler et al. [2].

Gas samples are admitted to the ionization chamber by means of an all-metal gas handling system, a 2-liter reservoir, and gold pinhole leak. Mass analysis is performed at a constant ion accelerating voltage of 2 kV by varying the magnetic field. The latter, set to focus a selected ion at the collector, is monitored by means of a Bell model 240 inoremental gaussmeter and recorded by a strip-chart recorder. Ion currents are measured with an electron multiplier, amplifier, and scaler. Maximum ion currents are of the order of 100 counts/s or 10^−17^ A. Within 20 Å (~0.2 eV) below threshold, maximum background currents are of the order of 1 count/s.

### 2.3. Photon Source

**Figure 4:**
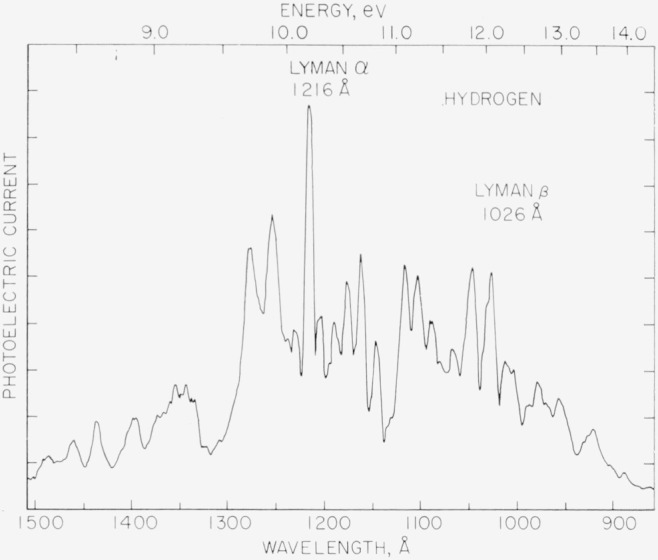
Tracing of the recorded photoelectric current from the photon detector as a function of wavelength for a d–c hydrogen discharge at a pressure of a few torr. Entrance and slits are 100*μ* and the scan rate is 100 Å/min.

Two types of lamps are used as photon sources. The first is a Hinteregger type similar to that described by Huffman, Tanaka, and Larrabee [6]. In operation, highest commercial purity cylinder gases without further purification enter through *G*_1_ and exit through the differential pumping ports. The discharge is excited by charging energy storage capacitors from an unregulated high-voltage d–c supply through a current-limiting resistor. For hydrogen at a pressure of a few torr, a steady operating current of 250 mA is obtained with about 600 V across the lamp. Figure 4 is a tracing of the photoelectric current from the photon detector as a function of the wavelength. The spectrum is scanned at the rate of 100 Å/min. Entrance and exit optical slits are 100 *μ* and no correction is made for the photoeletric efficiency of the detector. The Lyman-*α* line is easily identified and the relatively broad energy range of 1500 to 900 Å (8 to 14 eV) shown here indicates the general usefulness of this source of radiation. However, the large and frequent changes in photon intensity with wavelength characteristic of the many-lines emission spectrum superimposed on the Lyman continuum is a definite disadvantage.

Contrary to the above, the well-known rare-gas continua [7, 8, 9] provide comparatively uniform intensity with wavelength, although over shorter energy ranges. A recent study [6] has given details of best conditions for producing the helium continuum using a d–c discharge.

**Figure 5:**
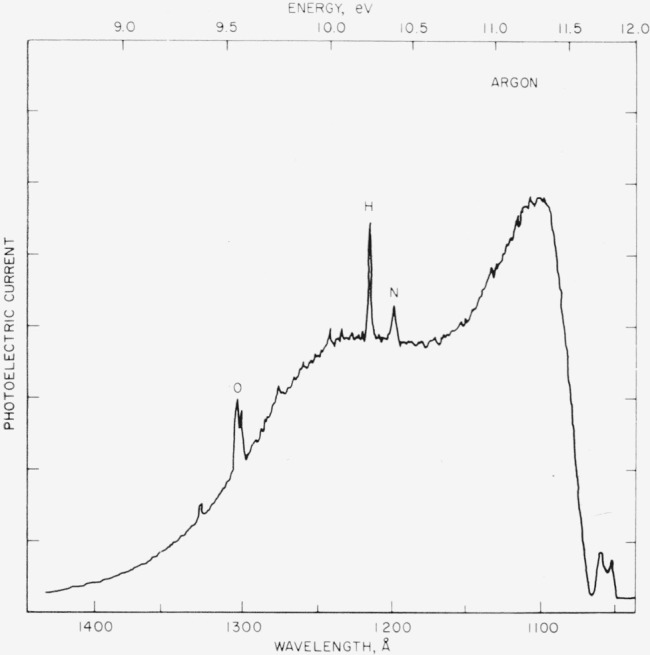
Tracing of the recorded photoelectric current of the photon monitor as a function of wavelength for the argon continuum at 400 torr. Slits are 100*μ* and the scan rate is 100 Å/min.

Tanaka and Zelikoff [10] first used microwave excitation in a windowless tube to obtain an emission continuum in xenon. Subsequent work [11] appeared so promising that a windowless microwave source was constructed for the present research. A 13-mm o.d. Vycor tube with 1-mm walls was sealed by means of epoxy resin into a hole drilled through the center of a water-cooled aluminum-alloy flange. The flanged end of the tube was terminated by a 0.3×6-mm slit in a thin stainless-steel sheet. The lamp was mounted with the slit end a few millimeters in front of the entrance optical slit of the monochromator. With argon flowing through the lamp at about 400 torr, the discharge is excited by a Raytheon model PGM-100, 2450 MHz, 250 to 800 W, cw generator. Figure 5 shows a tracing of the continuum recorded for 100-*μ* slits at a rate of 100 Å/min. The useful range in the present experiments extends from 1400 to 1070 A (8.8 to 11.6 eV). Small impurity peaks are usually observed and serve as convenient fiducial points for calibration of the wavelength scale. The stability of the lamp as measured by the photon intensity at any point on the continuum was usually better than ±1 percent at least during the time required to obtain the ion count.

**Figure 6:**
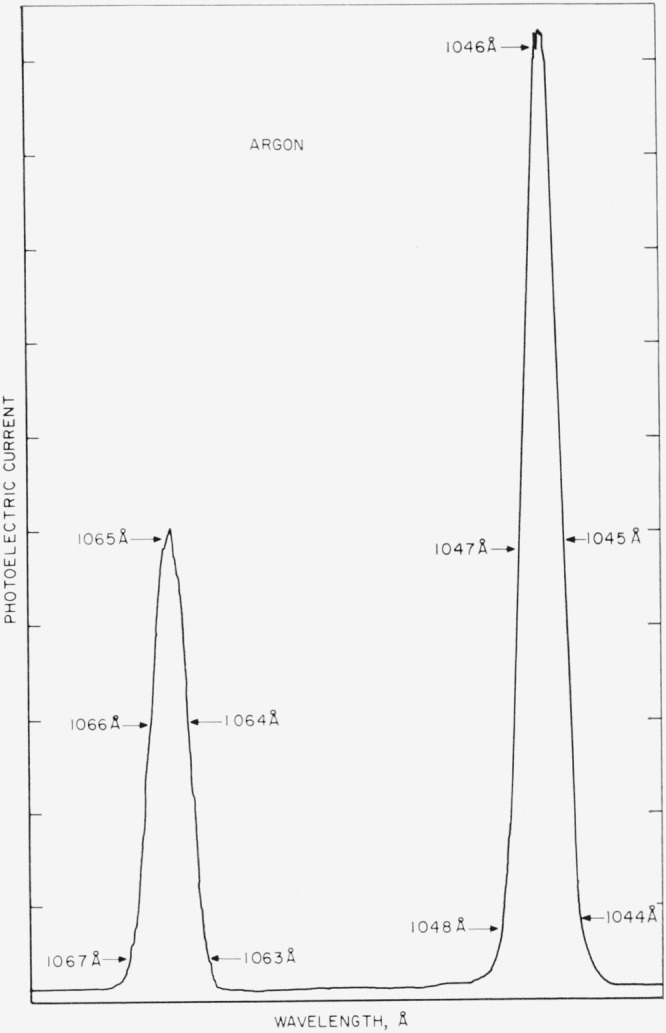
Tracing of the photoelectric current of the photon monitor as a function of wavelength for the argon doublet resonance lines at low pressure (a few torr). The slits are 100*μ* and the scan rate is 10 Å/min. The wavelength scale is uncorrected.

Figure 6 shows a tracing of the argon doublet resonance lines at 1066.7 and 1048.2 A obtained at low pressure (a few torr) to check the resolving power of the monochromator. The lines are scanned at a rate of 10 Å/min using 100-*μ* slits. The apparent half-width of the lines is about 2 Å which is consistent with a resolving power of 1.7 Å calculated from the slit dimensions. The actual line width, of course, is much narrower.

### 2.4. Photon Detector

Initial measurements were made using a sodium salicylate-coated photomultiplier tube [12]. However, the quantum efficiency of the coating was apparently affected by exposure to hydrocarbon samples or residual pumping vapors and it was discarded in favor of a photoelectric detector. The cathode of the latter was made from a tungsten^4^ sheet 10×40×0.1 mm taken from the laboratory stock and cleaned chemically. The anode was several 0.1-mm Nichrome wires mounted close to the surface and biased +45 V with respect to the cathode. The photoelectrons leaving the cathode were measured by means of a vibrating reed electrometer and recording potentiometer. The detector was enclosed in an open-mesh grid to shield it from the ion accelerating field in the ion source. Nevertheless, there remained the possibility of accelerating photoelectrons into the ionization chamber. This was shown to be of negligible importance by changing the field penetration, and introducing auxiliary electric and magnetic deflecting fields.

Although the quantum efficiency of photoelectron emitters varies with the wavelength, the general form of the function is known for many metals [1, 13]. Alternatively, the function may be approximated from an assumed or measured photoionization efficiency curve. Previous workers have used the photoionization of NO for this purpose [2]. In the present work, the relative quantum yield was assumed to follow the curves for untreated tungsten [14, 15]. In addition, the absolute yield at two wavelengths was estimated (probably within a factor of 2) by the following separate experiment: A krypton resonance lamp similar to that described for xenon by Wilkinson and Tanaka [11] and the photon detector used in this work were mounted at opposite ends of a cell containing parallel-plate electrodes for ion measurement [16]. With the cell evacuated, light from the resonance lamp operating at an arbitrary intensity was allowed to strike the detector and the photoelectric current was measured. Without disturbing the lamp, the cell was filled with NO under conditions of nearly total photon absorption [16, 17,]. Thus, the observed ion current between the electrodes was taken as a measure of the total number of photons entering the cell. The resultant nominal ratio of electrons/photon was one electron for 10^3^ photons. From the above measurement it was also possible to estimate that with a bandwidth of 2 Å, a maximum photon flux of 10^12^ quanta/s was produced at the entrance slit of the monochromator with the present windowless photon sources. Furthermore, with the same resonance lamp in place of the regular photon source of the monochromator, it was observed that the maximum photon flux through the ionization chamber was of the order of 10^10^ quanta/s.

### 2.5. Experimental Procedure

Briefly, a photoionization efficiency curve is obtained as follows: The photon source is excited and allowed to run for several minutes. The photoelectric current-versus-wavelength curve is obtained for the emission spectrum of the source, noting the apparent wavelengths of identifiable lines. Using a photon energy well above ionization threshold, the ion beam is focused at the ion collector. The grating is then turned to about 20 Å below threshold for the first observation, taking about 100 counts per point up to threshold. Usually 5000 or more counts are taken for each point above threshold. Thus the probable counting error of each point in this region is about 1.4 percent. The photon intensity and magnetic field strength are continuously monitored and recorded. The sample pressure is noted periodically and a correction for pressure drop in the sample reservoir is applied to the final data.

### 2.6. Materials

Ordinary acetylene, with a purity of 99.6 percent, as stated by the supplier, was obtained from the Matheson Company. Ordinary benzene was research-grade material from the laboratory stock. Acetylene-*d*_2_ and benzene-*d*_6_ were obtained from Merck, Sharpe, and Dohme, Montreal and had a stated isotopic purity of 99 atom percent D. Mass spectrometric analysis confirmed this value and indicated a comparable chemical purity.

## 3. Results and Discussion

### 3.1. Acetylene

**Figure 7:**
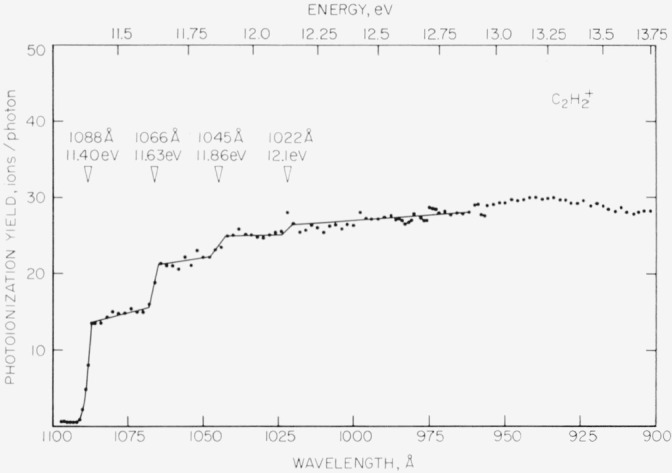
Photoionization efficiency curve for the *C*_2_*H*_2_^+^ ion of acetylene obtained by means of the hydrogen discharge as a photon source. Thresholds of ionization are indicated in angstroms and electron volts. All photoionization yields are in arbitrary units.

**Figure 8:**
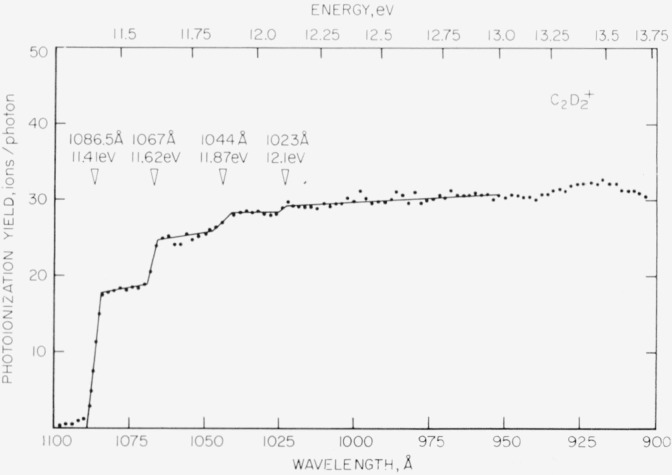
Photoionization efficiency curve for the *C_2_D_2_^+^* ion of acetylene-d_2_ obtained in the same manner as figure 7.

Photoionization efficiency curves for the C_2_H_2_^+^ and the C_2_D_2_^+^ ions of acetylene and acetylene-*d*_2_ obtained by means of the hydrogen discharge as a photon source are shown in figures 7 and 8, respectively. The photoionization yield (ions/photon) is plotted as a function of the wavelength in angstroms. The equivalent energy in electron volts is also given. The more fundamental property, the photoionization cross section, could not be determined under the conditions of these experiments. However, Nicholson [18] has shown that even for moderate absorption the resulting error in plotting the photoionization yield is small.

The first sharp rise in both curves is interpreted as the onset for the 0–0 transition for the lowest energy electron. Following Nicholson, the point of steepest ascent is chosen as the ionization energy. For acetylene, the arithmetic mean of five determinations of the first ionization energy is 1087.0 ±0.6 Å or 11.406 ±0.006 eV where the uncertainty is the computed probable error for a single determination. For acetylene-*d*_2_, the analogous values are 1086.0 ±0.6 Å or 11.416 ±0.006 eV.

Two Rydberg series in the vacuum ultraviolet absorption spectrum of acetylene were identified by Price [19]. Both series lead to an ionization energy of 11.41 eV. An identical value is reported by Watanabe [20, 21] using photoionization without mass analysis.

An ionization potential of 11.40 V for C_2_H_2_ was reported by Lossing, Tickner, and Bryce [22] using an electron-impact method. These authors also reported the value of 11.39 V for I(C_2_D_2_). However, they considered the ionization potentials of the isotopic molecules to be indistinguishable within the limits of their measurement (ca 0.1 V). From the present measurements it is apparent that the ionization energy of C_2_D_2_ is greater than that of C_2_H_2_ by about 0.01 eV as is expected from differences in zero-point energy.

The general shapes of the ionization efficiency curves for the two molecules are similar. In both cases, the first onset is followed at nearly uniform intervals by several well-defined onsets. For C_2_H_2_^+^, the intervals are approximately 0.23 eV or 1855 cm^−1^. For C_2_D_2_^+^, the intervals are not entirely consistent but appear to average about 0.22 eV, or 1775 cm^−1^. These ionization potentials are summarized in table 1.

Wilkinson [23] has obtained high resolution absorption spectra for acetylene and acetylene-*d*_2_ in the vacuum ultraviolet. Four electronic transitions were identified. One of these, designated 3R, is of a Rydberg type and probably involves a linear upper state. The *v*_2_ (carbon-carbon stretching frequency) of the 3R state was found to be 1848 cm^−1^ (0.23 eV) for C_2_H_2_ and 1720 cm^−1^ (0.21 eV) for C_2_D_2_. The agreement with the vibrational intervals shown in the figures and in table 1 is very satisfactory. Furthermore, the relative intensities of the vibrational steps observed in the present work are entirely consistent with Wilkinson’s observations. The simple vibrational structure and the apparently equally spaced vibrational levels indicate a linear ground state of the ion as suggested by Wilkinson.

The ground-state electronic configuration of acetylene can be expressed [24] as:

$$C_{2}H_{2}:\;{\left(\sigma_{g}1s_{c}\right)}^{2}{\left(\sigma_{u}1s_{c}\right)}^{2}{\left(2\sigma_{g}\right)}^{2}{\left(2\sigma_{u}\right)}^{2}{\left(3\sigma_{g}\right)}^{2}{\left(1\pi_{u}\right)}^{4},{\;}^{1}\Sigma_{g}{\;}^{+}.$$

It is very probable that the ionization energy of the 1*π_u_* electron is 11.41 eV. However, the ionization energy of the 3*σ_g_* electron is still a matter of conjecture. Recent calculations [25] result in an approximate value of 18.6 eV for the orbital energy of the 3*σ_g_* electron. An electron impact study [26] using simulated conditions of a monoenergetic electron beam has not confirmed that value. However, Lindhohn [27], using charge exchange, has observed just two electronic states of acetylene: one appearing at 11.4 eV and the other at about 15.5 eV with a remarkable gap in the distribution function between 12 and 15.5 eV. From the present work it is only concluded that no optically, allowed transition occurs at wavelengths above 900 Å ( $E\bar{<}14$ eV) and that the ionization energy of the 3*σ_g_* electron is at least 14 eV. Furthermore, in the absence of any suggestion of autoionized Rydberg terms it is probable that the orbital energy is several electron-volts above 14 eV.

### 3.2. Benzene

**Figure 9:**
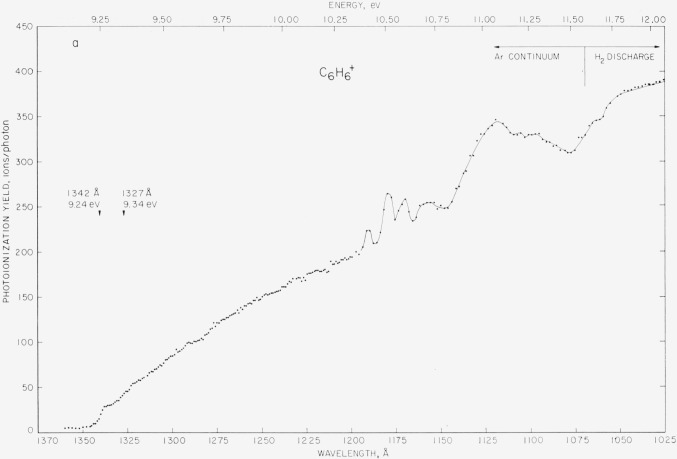

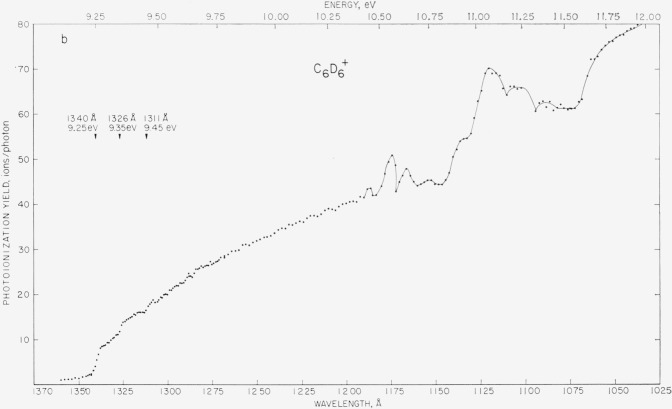
*a*, *b*. Ionization efficiency curves for the *C*_6_*H*_6_^+^ ion of benzene and the *C*_6_*D*_6_^+^ ion of benzene-*d*_6_ obtained by the use of the argon continuum and the hydrogen emission spectrum. Threshold energies for the ground-state ions are indicated.

Photoionization efficiency curves for the C_6_H_6_^+^ and the C_6_D_6_^+^ ions of benzene and benzene-*d*_6_ are shown in figure 9. The photon source for wavelengths above 1070 Å is the argon continuum. The hydrogen emission spectrum is used for wavelengths between 1070 and 900 Å. Although not shown beyond 1035 Å, the curves are continuous, slowly rising, and essentially smooth to 900 Å.

**Figure 10:**
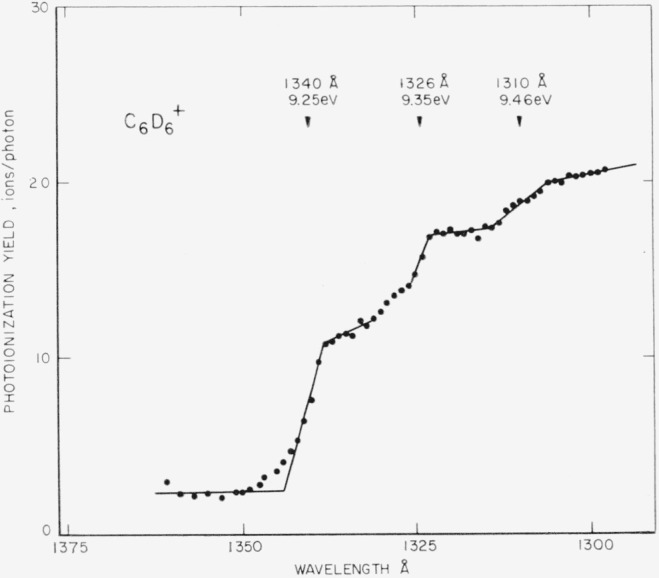
Enlarged-scale plot of the initial portion of an ionization efficiency curve for the *C*_6_*D*_6_^+^ ion showing the vibrational intervals of the ground state of the ion.

For the first ionization energy of benzene, the arithmetic mean and computed probable error of three determinations are 1341.5 ± 1.0 Å, or 9.24_2_ ±0.01 eV. For benzene-*d*_6_, the values are 1341.0 ±1.0 A, or 9.24_5_ ±0.01 eV. In both cases, the first onset is followed by several onsets ascribed to vibrational levels of the ground-state ion. Figure 10 shows an enlarged-scale plot of the initial portion of the curve for C_6_D_6_^+^. Although less well defined than in the case of the acetylenes, the quantum intervals for the benzenes are estimated to be 0.1 eV (~800 cm^−1^) and in the present experiments are apparently equal for the two isotopic molecules.

Rydberg series in benzene were first analyzed by Price and Wood [28]. More recently, Wilkinson [29] obtained high-resolution absorption spectra of benzene and benzene-*d*_6_ and identified four Rydberg series all converging to the same ionization limits: 9.247 ±0.002 eV (C_6_H_6_) and 9.251 ±0.002 eV (C_6_D_6_). These were assigned to the ionization of the lowest energy *π* electron. The present measurements are in good agreement with these and with recent photoionization measurements made without mass analysis [29].

The general shapes of both curves are quite similar. A Rydberg term with well-defined vibrational structure is observed in the region of 1180 Å (C_6_H_6_) and 1175 A (C_6_D_6_). The “peak” shapes indicate autoionization or predissociation from these levels. A less well-defined term or terms appears in the region of 1120 Å leading to the second ionization threshold at 1075 Å (11.5_3_ eV) for C_6_H_6_ and 1070 Å (11.5_9_ eV) for C_6_D_6_. These observations are generally consistent with the recent measurements by Tanaka and coworkers [30] and the suggested assignment of the second ionization energy to an electron from an (*a*_2_*_u_*) *π*-electron molecular orbital. The general features of an absorption curve of C_6_H_6_ reported by Goto [31] also show similarities with the present results.

From the second ionization potential (11.5 eV) and the usual Rydberg equation, series members leading to that ionization potential are calculated: one to appear at about 1175 Å (10.5 eV) and the other to appear very close to the first ionization limit. Both members have been observed by Price and Wood [28]. Wilkinson [29] reports a strong absorption doublet for benzene (1342.5 Å and 1341.5 Å) and for benzene-*d*_6_ (1335.4 Å and 1338.2 Å) which are very close to the onset of ionization. As no autoionization peaks are observed at the onset of ionization in the present work, it may be concluded that these transitions are to a neutral molecule rather than to the molecule ion.

Electron impact studies of benzene [32, 33] have given indications of several electronic states above the ground state of the ion. At present, the only firmly established onset would appear to be that of the (*a*_2_*_u_*) *π*-electron at 11.5 eV.

## Figures and Tables

**Table 1:** Summary of ionization energies for acetylene and acetylene-*d*_2_

Orbital and vibrational transition	Ionization energy	
	C_2_H_2_	C_2_D_2_
		
	*eV*	*eV*
1*π_u_* 0–0	a11.406 ±0.006	a11.416 ±0.006
0-1	b11.63 ±0.05	b11. 62 ±0.05
0-2	11.86 ±0.05	11.87 ±0.05
0-3	12.1 ±0.1	12.1 ±0.1

a Arithmetic mean and computed probable error for five independent measurements.

b Arithmetic mean and maximum deviation from the mean.

## Footnotes

1 Figures in brackets indicate the literature references at the end of this paper.

2 A brief announcement of this work appears in J. Chem. Phys. 40, 2034 (1964).

3 1 torr-1/760 atm-133.32 newtons-meter^−2^ (N/m^2^).

4 In preliminary experiments, cathodes were made of a variety of metals including rhenium, platinum, nichrome, and gold. No significant advantages over tungsten were observed.
